# Genetic Polymorphism of the Kinesin-Like Protein *KIF1B* Gene and the Risk of Hepatocellular Carcinoma

**DOI:** 10.1371/journal.pone.0062571

**Published:** 2013-04-25

**Authors:** Zhi-Chao Wang, Qiang Gao, Jie-Yi Shi, Liu-Xiao Yang, Jian Zhou, Xiao-Ying Wang, Ying-Hong Shi, Ai-Wu Ke, Guo-Ming Shi, Zhen-Bin Ding, Zhi Dai, Shuang-Jian Qiu, Jia Fan

**Affiliations:** 1 Liver Cancer Institute, Zhongshan Hospital, Fudan University, Shanghai, P. R. China; 2 Key Laboratory of Carcinogenesis and Cancer Invasion, Ministry of Education, Fudan University, Shanghai, P. R. China; 3 Institute of Biomedical Sciences, Fudan University, Shanghai, P. R. China; MOE Key Laboratory of Environment and Health, School of Public Health, Tongji Medical College, Huazhong University of Science and Technology, China

## Abstract

**Background:**

Frequent deletions of the kinesin-like protein gene 1B (KIF1B) have been reported in neural tumors. Recently, a genome-wide association study revealed an association between polymorphisms in the KIF1B gene and the risk of hepatocellular carcinoma (HCC), and several case-control studies have further investigated this relationship. However, these studies have yielded controversial results. We therefore performed a meta-analysis to derive a more precise estimation of the association between the KIF1B gene polymorphisms and HCC risk.

**Methodology/Principal Finding:**

PubMed, EMBASE, the ISI Web of Science and the CNKI databases were systematically searched to identify relevant studies. A total of 5 studies containing 13 cohorts with 5,773 cases and 6,404 controls were included. Odds ratios (ORs) with corresponding 95% confidence intervals (CIs) were used to assess the strength of the associations. Subgroup analyses were conducted based on ethnicities, sample sizes and quality scores. Overall, the G allele at rs17401966 of the KIF1B gene was associated with a significantly decreased risk for HCC (OR  = 0.81, 95%CI: 0.70–0.93; P = 0.003). Furthermore, subgroup analyses showed that the G allele at rs17401966 of the KIF1B gene significantly reduced the risk for HCC in Chinese cohorts (OR  = 0.76, 95%CI: 0.64–0.90; P = 0.002), large-sample-size cohorts (OR  = 0.80, 95%CI: 0.73–0.88, P<0.01) and high-quality cohorts (OR  = 0.78, 95%CI: 0.71–0.87, P<0.01). However, no significant associations were found in small-sample-size cohorts, studies with low-quality scores and when excluding the cohorts from the study reporting the original discovery.

**Conclusion/Significance:**

These findings demonstrate that the presence of the G allele at rs17401966 of the KIF1B gene may decrease the risk for HCC and suggest that KIF1B may play a critical role in the development of HCC. High-quality studies with larger sample sizes and different ethnic populations will be of great value to further confirm these findings.

## Introduction

Hepatocellular carcinoma (HCC) is the sixth most prevalent cancer worldwide and the third most frequent cause of cancer-related death [1]. Globally, there are approximately 750,000 new cases reported each year, and half of them occur in China [2]. The etiological importance of chronic infection with the hepatitis B virus (HBV) and the hepatitis C virus (HCV) in HCC has been well established [3]. In addition, tobacco smoking, alcohol drinking and aflatoxin exposure were also shown to be risk factors for HCC [4]–[6]. However, only a minority of individuals who are at risk will develop HCC, indicating that the theory of conventional risk factors can only explain part of the pathogenesis of HCC. Notably, there is considerable evidence strongly supporting the importance of a genetic predisposition in HCC patients [7].

During the last several decades, hypothesis-based research has revealed a number of genetic predispositions associated with HCC [7]. Recently, genome-wide association studies (GWAS), a hypothesis-free approach, is now being used to study HCC [8], giving us better insight into the underlying mechanisms of hepatocarcinogenesis and providing information beyond those derived from logical analysis. Based on the results of genetic association studies, several loci are believed to be associated with HCC, such as MICA [9], HLA-DQA1/DRB1 [10] and SATA4 [11].

Over the past decades, kinesin-like factor 1 B (KIF1B) has been explored extensively as a kinesin superfamily member. The members of this family are responsible for the transport of organelles, vesicles, protein complexes and RNA to specific destinations [12]. KIF1B encodes two alternatively spliced isoforms, KIF1Bα and KIF1Bβ. Both isoforms form homodimers and transport mitochondria and synaptic vesicle precursors, respectively [13]. It has been postulated that by regulating mitochondria transportation, KIF1Bα might prevent energy expenditure cancer cells and suppress cancer growth. Likewise, KIF1Bβ can induce apoptosis by acting downstream of EglN3 prolyhydroxylase, which may lead to inhibition of malignant transformation and progression [14]. As such, KIF1B may function as a tumor suppressor. Although somatic and germline loss-of-function mutations in KIF1B have been reported in neural tumors [14]–[17] and multiple sclerosis [18], a GWAS performed by Zhang et al. is the first investigation to report this association in HCC [19]. Since then, considerable efforts have been devoted to validating this relationship in various populations. However, the existing studies have yielded inconsistent or conflicting results. These controversies may be partly ascribed to different ethnicities, small sample sizes, various levels of quality, false-positive results and publication biases. We therefore performed a meta-analysis of the published studies to clarify this inconsistency and to establish a comprehensive picture of the relationship between KIF1B polymorphisms and HCC.

## Materials and Methods

### Literature Search Strategy and Selection Criteria

A systematic review and meta-analysis were performed according to the guidelines of PRISMA (Preferred Reporting Items for Systematic Reviews and Meta-Analysis) [20]. We searched PubMed (1966 through Dec 21, 2012), Web of Science (1993 through Dec 21, 2012), EMBASE (1988 through Dec 21, 2012) and CNKI (1949 through Dec 21, 2012) without language limitations, using the following search algorithm: (“hepatocellular carcinoma” or “hepatocellular neoplasm” or “liver cancer” or “HCC”) and (“kinesin-like protein” or “kinesin-like protein 1 B” or “KIF1B” or “KIF”) and (“polymorphism” or “variation” or “susceptibility”). Additionally, all of the references cited in the retrieved articles were reviewed to identify additional studies.

Studies were considered for inclusion in the meta-analysis if they met the following criteria: (i) evaluated the association of KIF1B gene polymorphisms and the risk of HCC, (ii) cohort-based or case-control studies, and (iii) presented sufficient data for calculating the odds ratios (ORs) with corresponding 95% confidence intervals (95%CIs). The major exclusion criteria were as follows: (i) review or comment articles, case-only studies or family-based studies, (ii) lack of availability of sufficient data for estimation of ORs with 95%CIs, and (iii) duplication of previous publications and replicated samples.

### Eligible Studies and Data Extraction

Two reviewers (Z.C. Wang and Q. Gao) screened the studies and extracted the raw data from all eligible publications independently. The following characteristics were collected using a data extraction form that included the first author's name, the year of publication, the cohort of the study, the ethnicity of the patients, the type and number of patients and controls with various genotypes, age at the time of the study, gender and genotyping method. Genotype distributions reported in percentages were converted to numbers. If allele frequencies were not given, they were calculated from the corresponding genotype distributions. For some studies [10], [19], [21], including those with cohorts of different ethnic populations, the data were collected separately whenever possible and the data sets were recognized as independent studies. To ensure the accuracy of the data extraction, the original data extraction was checked by J.Y. Shi. Disagreements were resolved by discussion among the authors.

### Statistical Methods

For the KIF1B gene, we estimated the HCC risk associated with the G allele of the KIF1B gene at rs17401966 in dominant, co-dominant and recessive genotypic models, compared with the A allele. The genotypic models were as follows: allele (G vs. A), co-dominant (GG vs. AA; GG vs. AG and AG vs. AA), dominant (GG+AG vs. AA), and recessive (GG vs. AG+AA). ORs with corresponding 95% confidence intervals (CIs) and P-values were calculated to estimate the strength of the association between the KIF1B polymorphisms and risk of HCC.

A Cochrane chi-square-based Q-test was performed to test the heterogeneity among studies or cohorts [22]. The *I* ^2^ tests were performed to assess the statistical heterogeneity, and the Q-statistic tests with P≤0.10 were used to define a significant degree of heterogeneity. If there was no heterogeneity (P>0.10), the fixed effects were calculated as the inverse variance-weighted average of the log OR. If there was substantial heterogeneity (P≤0.10), random effects were calculated. The 95% CIs were calculated using Woolf's method [23]. In addition, the sources of heterogeneity were investigated by stratified meta-analyses based on ethnicities, sample sizes (No. of cases ≥200 or <200) and quality scores (Score ≥7 or <7). All probability values were two-sided, and values of P<0.05 were considered statistically significant. Sensitivity analysis was performed to assess the stability of the results of the meta-analysis. Funnel plots were performed to allow visual inspection of the results of Egger's linear regression test [24]. Statistical analyses were conducted using validating Review manager Version 5.1 (Copenhagen, The Nordic Cochrane Centre, The Cochrane Collaboration, 2011).

## Results

### Characteristics of Studies

The combined search yielded 21 references. Eventually, 16 studies were excluded based on the inclusion/exclusion criteria (Figure S1), resulting in a total of 5 studies containing 13 cohorts [10], [19], [21], [25], [26]. The quality of the cohorts was measured according to the score scale for a randomized controlled association studies proposed by Clark et al. [27]. The characteristics of the included studies and cohorts are summarized in Table S1. Overall, there were 5 studies containing 13 cohorts with 5,773 cases and 6,404 controls, while in the Chinese subgroups, there were 5,103 cases and 4,794 controls.

### Meta-analysis

We perform the meta-analysis of the included studies in three stages. In the first stage, we analyzed the pooled data including populations from China, Japan, Korea and Saudi Arabia, irrespective of ethnicity. In the second stage, we stratified thirteen cohorts by their ethnicities. Of the thirteen cohorts, there were nine Chinese cohorts, two Japanese cohorts, one Korean cohort and one Saudi Arabian cohort. We only performed subgroup analysis in the Chinese cohorts because the other three ethnicities did not meet the PRISMA criteria that a meta-analysis usually applies to more than 3 original data sets [20]. In the third stage, we tried to verify this association in non-reported cohorts to avoid reporting and selection biases. Accordingly, 5 cohorts of the discovery study were excluded from the meta-analysis at this stage.

### Association of KIF1B polymorphism with HCC among all 13 cohorts

**Figure 1:**
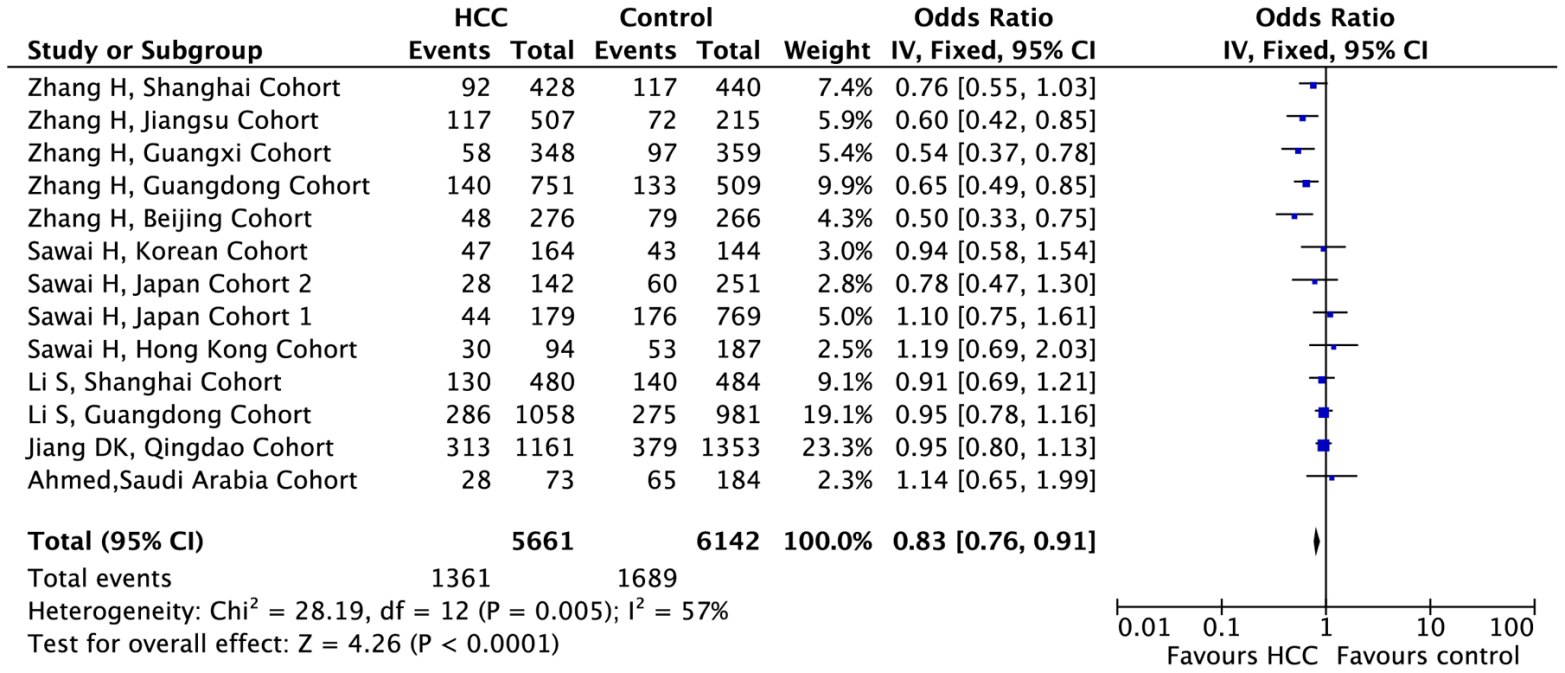
Forest plot presenting the meta-analysis of KIF1B polymorphisms and the susceptibility to hepatocellular carcinoma under the genotypic model G vs. **A in the 13 cohorts.** The horizontal lines represent 95% confidence intervals for estimating the outcome of the G allele versus the A allele in the meta-analysis. (▪) Overall estimates of the effects.

In the pooled analysis of 13 cohorts, a significant allelic association was recorded under a random-effect model, with OR  = 0.81 (95% CI, 0.70–0.93; P<0.01), indicating that the G allele was associated with a decreased HCC risk compared to the A allele (Figure 1). The heterogeneity of this pooled analysis was statistically significant, with P = 0.005. A significant genotypic association with the risk of HCC was observed in the pooled studies under co-dominant, dominant and recessive genotype models (Table 1). Under the co-dominant genotype model (GG vs. AG), the lowest heterogeneity was observed (P = 0.15) with the highest effect (OR  = 0.70). The subgroup analyses on the basis of sample size showed the decreased HCC risk in large-sample-size cohorts (OR  = 0.80; 95%CI, 0.73–0.88; P<0.01) rather than in small-sample-size cohorts (OR  = 1.02, 95%CI: 0.82–1.27, P = 0.85). Subgroup analyses were also performed according to the quality scores. The results indicated that the G allele was associated with a decreased HCC risk in studies with high-quality scores (OR  = 0.78, 95%CI: 0.71–0.87, P<0.01), but not in those with low-quality scores (OR  = 0.95, 95%CI: 0.82–1.11, P = 0.53).

**Table 1:** Allelic and genotypic meta-analysis of the KIF1B polymorphism at rs17401966 in all cohorts under alternative genetic models.

Allele/ genotype	HCC	Control	HCC vs. Control			Heterogenity	
			OR	CI	P	*I^2^*	P
rs17401966							
G vs. A	5661	6142	0.81	[0.70, 0.93]	0.003	57%	<0.01
GG vs. AA	1997	2074	0.53	[0.34, 0.83]	0.005	69%	<0.01
GG vs. AG	1088	1483	0.70	[0.55, 0.89]	0.004	33%	0.15
AG vs. AA	2835	3089	0.71	[0.64, 0.80]	<0.001	47%	0.05
GG+AG vs. AA	2962	3324	0.72	[0.59, 0.86]	<0.001	65%	<0.01
GG vs. AG+AA	2962	3324	0.59	[0.40, 0.87]	0.008	62%	<0.01

HCC, hepatocellular carcinoma; CI, confidence interval; OR, odds ratio.

### Association of KIF1B polymorphism with HCC in the Chinese subgroups

**Figure 2:**
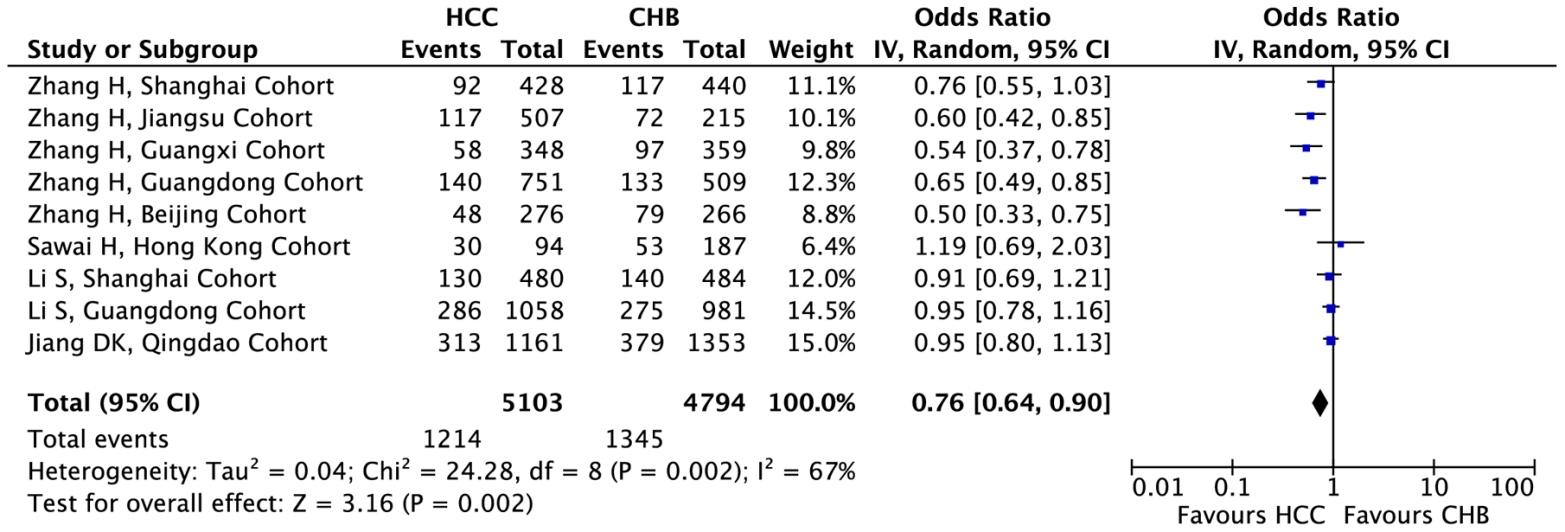
Forest plot presenting the meta-analysis of KIF1B polymorphisms and the susceptibility to hepatocellular carcinoma under the genotypic model G vs.A in the Chinese subgroups. The horizontal lines represent 95% confidence intervals for estimating the outcome of the G allele versus the A allele in the meta-analysis. (▪) Overall estimates of the effects.

When stratifying the pooled data, only the Chinese subgroups met the criteria for subgroup analysis. The significant associations were detected under dominant (GG+AG vs. AA: OR  = 0.60, 95%CI: 0.53–0.68), co-dominant (GG vs. AA: OR  = 0.39, 95%CI: 0.23–0.65; GG vs. AG: OR  = 0.59, 95%CI: 0.44–0.79; AG vs. AA: OR  = 0.64, 95%CI: 0.57–0.73) and recessive (GG vs. AG+AA: OR  = 0.45, 95%CI: 0.28–0.72) genotypic models (Figure 2 and Table 2). However, high heterogeneity was also observed in dominant (GG+AG vs. AA: *I^2^* = 45%, P = 0.01) and recessive (GG vs. AG+AA: *I^2^* = 61%, P = 0.03) genotypic models. Notably, a low heterogeneity and a high effect were also detected in co-dominant models (GG vs. AG; AG vs. AA). Compared with the heterogeneity and significance in all the 13 cohorts, the heterogeneity in the Chinese subgroups did not decrease considerably, except in the co-dominant model (AG vs. AA: *I^2^* = 47%, P = 0.03 in all the 13 cohorts vs. *I^2^* = 13%, P = 0.33 in Chinese subgroups), while the effects and significance levels all slightly increased within the co-dominant (GG vs. AA: OR = 0.53, P = 0.005 in all the 13 cohorts vs. OR  = 0.39, P<0.001 in Chinese subgroups; GG vs. AG: from OR  = 0.70, P = 0.004 in all 13 cohorts to OR  = 0.59, P<0.001 in Chinese subgroups; AG vs. AA: OR  = 0.71, P<0.001 in all the 13 cohorts vs. OR  = 0.64, P<0.001 in Chinese subgroups), dominant (GG+AG vs. AA: OR  = 0.72, P<0.001 in all the 13 cohorts vs. OR  = 0.60, P<0.001 in Chinese subgroups) and recessive (GG vs. AG+AA: OR  = 0.59, P = 0.008 in all the 13 cohorts vs. OR  = 0.45, P<0.001 in Chinese subgroups) genotypic models.

**Table 2:** Allelic and genotypic meta-analysis of KIF1B polymorphism at rs17401966 in Chinese cohorts under alternative genetic models.

Allele/genotype	HCC	Control	HCC vs. Control			Heterogenity	
			OR	CI	P	*I^2^*	P
rs17401966							
G vs. A	5103	4784	0.76	[0.64, 0.90]	0.002	67%	<0.01
GG vs. AA	1628	1181	0.39	[0.23, 0.65]	<0.001	67%	0.01
GG vs. AG	862	946	0.59	[0.44, 0.79]	<0.001	40%	0.14
AG vs. AA	2316	1825	0.64	[0.57, 0.73]	<0.001	13%	0.33
GG+AG vs. AA	2404	1976	0.60	[0.53, 0.68]	<0.001	45%	0.01
GG vs. AG+AA	2404	1976	0.45	[0.28, 0.72]	<0.001	61%	0.03

HCC, hepatocellular carcinoma; CI, confidence interval; OR, odds ratio.

### Association of KIF1B polymorphism with HCC excluding the discovery study

To decrease the reporting bias and increase reliability, we conducted the meta-analysis excluding the discovery study. A meta-analysis was performed in the remaining cohorts after excluding the discovery cohorts (Figure S2 and Table S2). No significance was detected under any genotypic models using a fixed-effects model. The heterogeneity under all genotypic models was quite low, with P values ranging from 0.62 to 0.95.

### Sensitivity Analyses and Publication Bias

Each cohort was excluded individually to investigate the influence of the individual data set on the pooled ORs. The exclusion of any single cohort did not alter the overall significance of the final decision, suggesting that the results were robust.

Funnel plots were calculated in each stage of the meta-analysis to allow for visual inspection of the results of Egger's test. The shape of the funnel plots for these polymorphisms was symmetrical (Figure S3), indicating that there were no publication biases in the studies of KIF1B polymorphisms.

## Discussion

Unbiased epidemiological investigations with large sample sizes of gene polymorphisms can provide insights into the relationship between candidate genes and diseases. Through this method, many associations between gene polymorphisms and the risk of several types of cancers have been detected [28], [29]. Recently, a GWAS first reported KIF1B polymorphisms at key loci associated with HCC [19]. Several studies were conducted to validate this finding, yielding controversial results. Herein, we present the first comprehensive meta-analysis to examine the relationship between KIF1B polymorphisms and HCC susceptibility. In total, the meta-analysis involved 5 studies containing 13 cohorts with 5,773 cases and 6,404 controls, which doubles the sample size in the original discovery study.

The positive association of the G allele at rs17401966 in KIF1B polymorphisms among all populations and in the subgroup of the Chinese population under co-dominant, dominant and recessive genotype models suggests that this variant could decrease HCC risk in these populations. Even though the heterogeneity among these studies and cohorts was relatively high, the association still existed under a random-effect model. Subgroup analyses revealed that cohorts with small sample sizes and low quality scores were sources of heterogeneity. In addition, the decreased heterogeneity and the increased significance in Chinese subgroups suggested that the high heterogeneity was also partly related to variations in ethnicity.

A meta-analysis of the cohorts while excluding those used in the previous discovery study showed no association between KIF1B polymorphisms at rs17401966 and HCC risk. This inconsistency might be attributed to the fact that there were heterogeneous population structures, considering that 4 different ethnicities were represented in the cohorts when the cohorts from the discovery studies were excluded, particularly the frequencies of the G allele varied between the Saudi Arabian cohort (P = 0.39) [25] and the Japanese cohort (P = 0.20) [21]. This remarkable difference in the frequency of the G allele among the different cohorts might due to distinct genetic backgrounds and environmental pressures [30]. Therefore, it is necessary to investigate the exact effect of ethnicity on the association between KIF1B polymorphisms and HCC risk. Meanwhile, the small sample sizes should also be taken into consideration. Compared with the discovery study [19], the sample sizes of the following studies [10], [21], [25], [26] were relatively small. Further studies including larger sample sizes across multiple ethnicities are needed before a comprehensive conclusion can be made.

KIF1B, located on chromosome 1p36.21, encodes a kinesin superfamily member involved in the transport of organelles and vesicles [31]. Previously, Midorikawa et al. reported that 1p36.21-36.22 contained a potential tumor suppressor gene involved in hepatocarcinogenesis [32]. Moreover, loss of heterozygosity at 1p36 has been identified in various human cancers [33]. In line with these results, our meta-analysis also indicated that KIF1B at this locus is a biologically plausible candidate for HCC susceptibility. As confirmation, KIF1Bα and KIF1Bβ, two isoforms of the KIF1B gene, have been proposed to have tumor inhibiting effects [14], [34]. Recently, Zhang R et al. further confirmed that KIF1B was a distinct genetic factor contributing to the progression from chronic hepatitis B virus infection to HCC [35]. However, the exact function of the KIF1B gene in HCC is unclear, and conditional knockout models may be necessary to further investigate the role of this protein in hepatocarcinogenesis.

Some limitations of our meta-analysis should be acknowledged when interpreting the results. First, the between-cohort heterogeneity was a major concern because obvious heterogeneity was detected in the overall analyses and Chinese subgroups. We explored several possible sources of heterogeneity, including ethnicity, sample sizes and quality scores. Fortunately, we did find that small sample sizes and low quality scores were sources for this heterogeneity. However, we cannot entirely rule out the possibility that other unknown confounding factors might have biased this finding. Therefore, the conclusion should be adopted conservatively. Second, some potential confounding factors, such as age, tobacco smoking, alcohol drinking and aflatoxin exposure, could not be assessed due to insufficient data. Third, the data on ethnicities other than Chinese were limited. Finally, the sample sizes were relatively small, especially in some subgroup analyses.

In conclusion, this study is the first meta-analysis to summarize the relationship between KIF1B gene polymorphisms and the susceptibility to HCC. Our results suggests that the G allele at rs17401966 in the KIF1B gene decreases the risk of HCC, providing additional data supporting the potential functional role of KIF1B in hepatocarcinogenesis, which needs to be authenticated through molecular and cellular approaches. Further studies with large sample sizes, different ethnic populations and the exclusion of other confounding factors are still required.

## Supporting Information

Figure S1 **Selection of the related studies.**
(TIF)

Figure S2 **Forest plot of association between KIF1B polymorphisms and HCC risk when excluding the discovery cohorts.**
(TIF)

Figure S3 **Funnel plot of the association between KIF1B polymorphisms and HCC risk in all cohorts.**
(TIF)

Table S1 **Characteristics of the studies and cohorts included in the meta-analysis.**
(DOC)

Table S2 **KIF1B polymorphisms in all cohorts when excluding the discovery study under alternative genetic models.**
(DOC)

Table S3 **PRISMA 2009 checklist of this systematic review and meta-analysis.**
(DOC)
