# Supplementary figures and images for: Genetic Polymorphism of the Kinesin-Like Protein KIF1B Gene and the Risk of Hepatocellular Carcinoma
Source: PLoS One. 2013 Apr 25;8(4):e62571. doi: 10.1371/journal.pone.0062571 (PMC3636275; doi:10.1371/journal.pone.0062571)

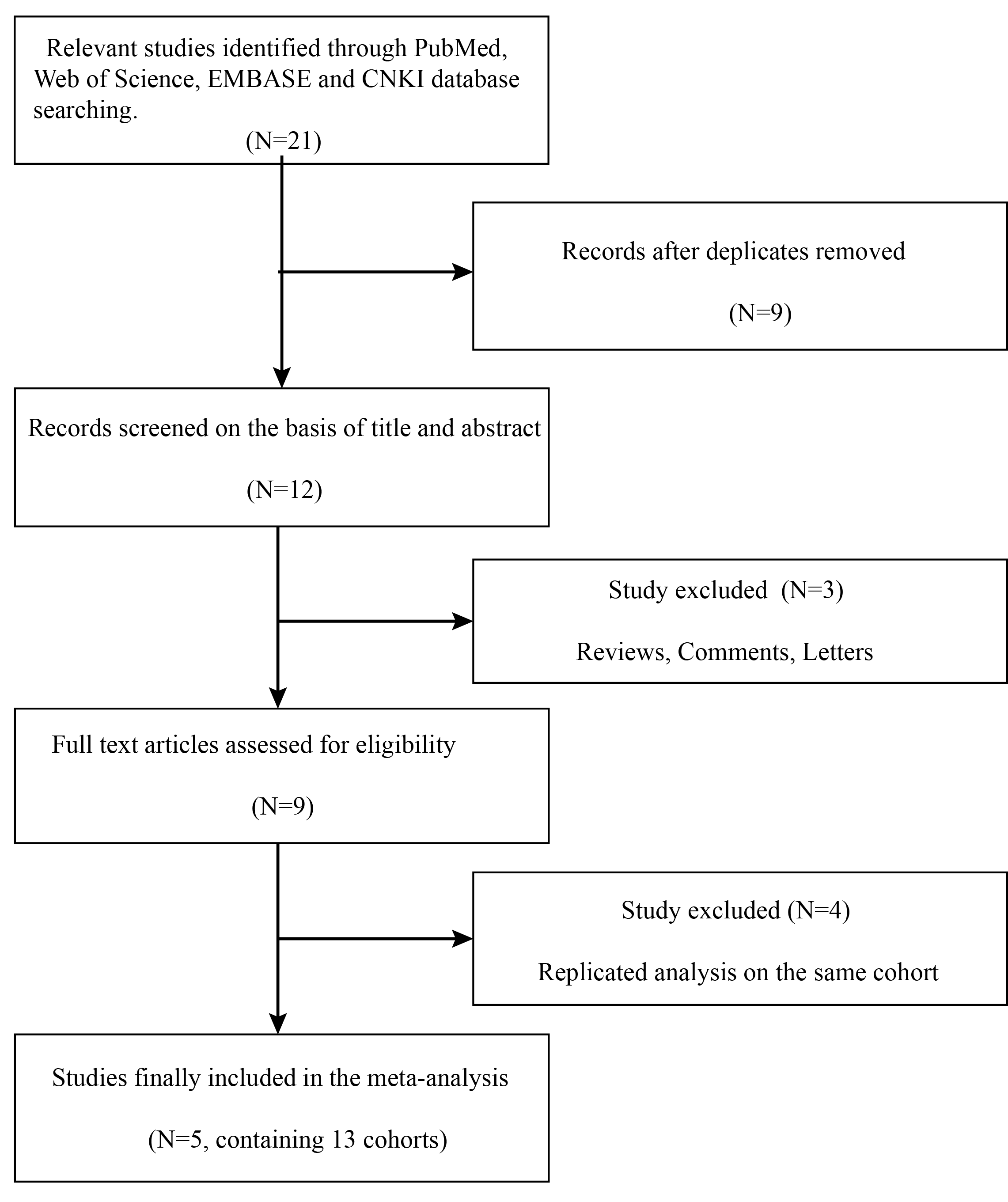

Supplement: Figure S1 — Selection of the related studies. (TIF) [file pone.0062571.s001.tif]

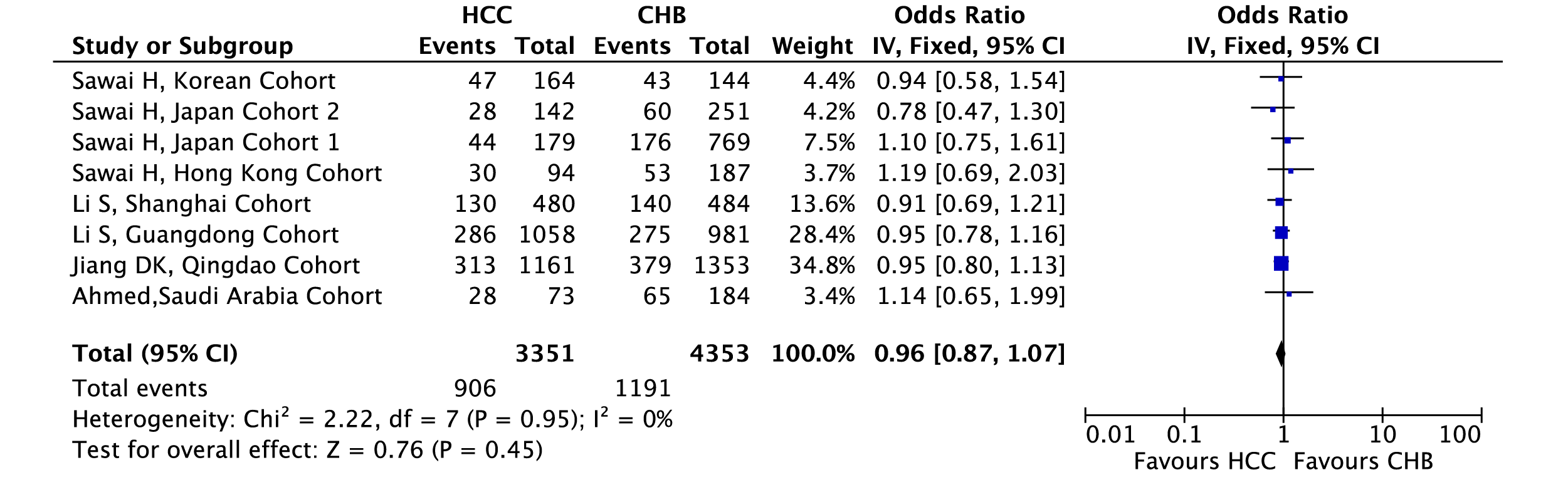

Supplement: Figure S2 — Forest plot of association between KIF1B polymorphisms and HCC risk when excluding the discovery cohorts. (TIF) [file pone.0062571.s002.tif]

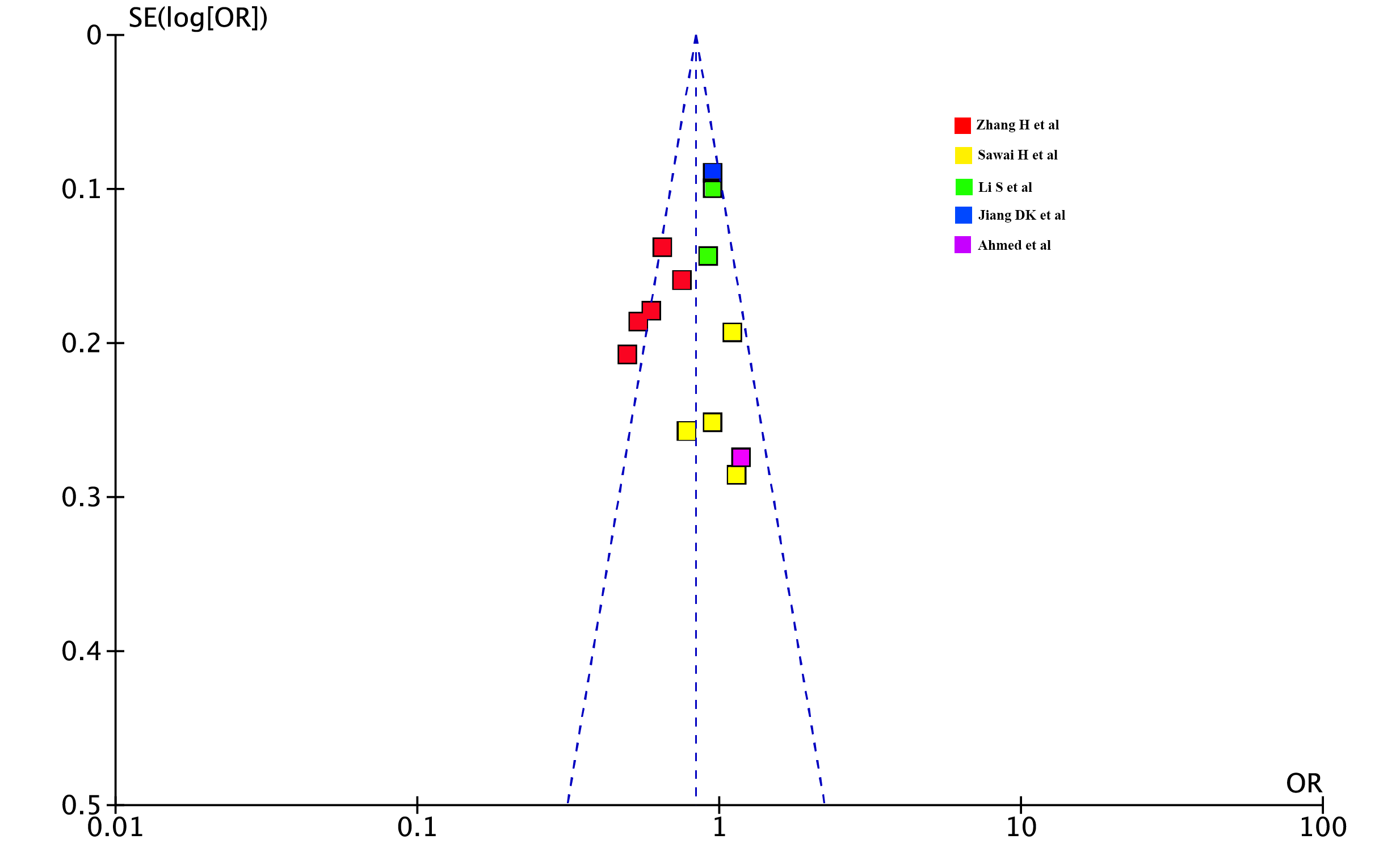

Supplement: Figure S3 — Funnel plot of the association between KIF1B polymorphisms and HCC risk in all cohorts. (TIF) [file pone.0062571.s003.tif]
