# Supplementary material for: Genetic Polymorphism of the Kinesin-Like Protein KIF1B Gene and the Risk of Hepatocellular Carcinoma
Source: PLoS One. 2013 Apr 25;8(4):e62571. doi: 10.1371/journal.pone.0062571 (PMC3636275; doi:10.1371/journal.pone.0062571)
Supplement: Table S1 — Characteristics of the studies and cohorts included in the meta-analysis. (DOC) [file pone.0062571.s004.doc]

**Table S1. Characteristics of the studies and cohorts included in the meta-analysis.**

HBV, Hepatitis B virus; HCC, Hepatocellular carcinoma; CHB, Chronic hepatitis B. “-” stands for unclear.

| **Name of Studies** | **Name of cohorts** | **No. of Case** | **No. of Control** | **Type of Case/**  **Control** | **Ancestry** | **Genotyping method** | **Quality**  **Scores** | **Age** | | | | **Male/Female ratio** | |
| --- | --- | --- | --- | --- | --- | --- | --- | --- | --- | --- | --- | --- | --- |
|  |  |  |  |  |  |  |  | **Case**  **Mean SD** | | **Control**  **Mean SD** | | **Case** | **Control** |
| Zhang H, 2010 [16] | Guangxi | 348 | 359 | HBV-related HCC/CHB | Chinese | Affymetrix 6.0 | 9 | 45.8 | 10.6 | 41.6 | 12.1 | 6.73 | 6.32 |
|  | Beijing | 276 | 266 | HBV-related HCC /CHB | Chinese | SNPstream | 8 | 55.9 | 9.1 | 55.5 | 12.2 | 6.07 | 5.49 |
|  | Jiangsu | 507 | 215 | HBV-related HCC /CHB | Chinese | Taqman | 8 | 52.7 | 10.8 | 52.9 | 11.2 | 3.78 | 4.38 |
|  | Guangdong | 751 | 509 | HBV-related HCC /CHB | Chinese | - | 7 | 49.3 | 11.6 | 48.1 | 11.4 | 6.59 | 5.28 |
|  | Shanghai | 428 | 440 | HBV-related HCC /CHB | Chinese | - | 7 | 50.6 | 9.1 | 51.2 | 8.5 | 7.39 | 6.72 |
| Li S, 2012 [23] | Guangdong | 1058 | 981 | HBV-related HCC /CHB | Chinese | - | 6 | 49.3 | 11.4 | 48.8 | 11.7 | 7.79 | 3.47 |
|  | Shanghai | 480 | 484 | HBV-related HCC /CHB | Chinese | - | 6 |  |  |  |  |  |  |
| Sawai H, 2012 [25] | Hong Kong | 94 | 187 | HBV-related HCC /CHB | Chinese | PCR | 7 | 58.0 | 10.5 | 56.9 | 8.3 | 5.71 | 5.67 |
|  | Japan 1 | 179 | 769 | HBV-related HCC /CHB | Japanese | Taqman | 7 | 62.0 | 9.4 | 54.7 | 13.5 | 4.26 | 1.76 |
|  | Japan 2 | 142 | 251 | HBV-related HCC /CHB | Japanese | Taqman | 7 | 61.3 | 10.2 | 56.2 | 10.9 | 4,46 | 1.51 |
|  | Korea | 94 | 187 | HBV-related HCC /CHB | Korean | Taqman | 7 | 52.2 | 8.9 | 37.3 | 11.3 | 3.46 | 3.50 |
| Ahmed A, 2012 [22] | Saudi Arabia | 255 | 403 | HBV-related HCC /CHB | Saudi Arabia | PCR | 6 | - | - | - | - | - | - |
| Jiang DK, 2012 [24] | Qidong | 1161 | 1353 | HBV-related HCC /CHB | Chinese | Affymetrix 6.0 | 8 | 51.6 | 10.4 | 48.4 | 11.1 | 3.41 | 2.10 |
| Total |  | 5773 | 6404 |  |  |  |  |  |  |  |  |  |  |
