# Supplementary material for: Genetic Polymorphism of the Kinesin-Like Protein KIF1B Gene and the Risk of Hepatocellular Carcinoma
Source: PLoS One. 2013 Apr 25;8(4):e62571. doi: 10.1371/journal.pone.0062571 (PMC3636275; doi:10.1371/journal.pone.0062571)
Supplement: Table S2 — KIF1B polymorphisms in all cohorts when excluding the discovery study under alternative genetic models. (DOC) [file pone.0062571.s005.doc]

| Allele/genotype | HCC | Control | HCC vs. Control | | | Heterogenity | |
| --- | --- | --- | --- | --- | --- | --- | --- |
|  |  |  | OR | CI | P | *I^2^* | P |
| rs17401966 | | | | | | | |
| G vs. A | 3351 | 4353 | 0.96 | [0.87, 1.07] | 0.45 | 0% | 0.95 |
| GG vs. AA | 423 | 1000 | 1.08 | [0.74, 1.64] | 0.68 | 0% | 0.62 |
| GG vs. AG | 275 | 630 | 0.62 | [0.74, 1.64] | 0.62 | 0% | 0.78 |
| AG vs. AA | 602 | 1438 | 0.98 | [0.80, 1.20] | 0.84 | 0% | 0.80 |
| GG+AG vs. AA | 652 | 1535 | 0.99 | [0.81, 1.20] | 0.90 | 0% | 0.72 |
| GG vs. AG+AA | 652 | 1535 | 1.09 | [0.75, 1.59] | 0.64 | 0% | 0.68 |

**Table S2. Allelic and genotypic meta-analysis of the KIF1B polymorphism at rs17401966 in all cohorts, excluding the discovery study, under alternative genetic models.**

HCC, hepatocellular carcinoma; CI, confidence interval; OR=odds ratio.
